# New Treatment for the Cognitive and Emotional Deficits Linked with Paclitaxel-Induced Peripheral Neuropathy in Mice

**DOI:** 10.3390/antiox11122387

**Published:** 2022-12-01

**Authors:** Ignacio Martínez-Martel, Xue Bai, Gerard Batallé, Olga Pol

**Affiliations:** 1 Grup de Neurofarmacologia Molecular, Institut d’Investigació Biomèdica Sant Pau (IIB SANT PAU), Sant Quintí 77-79, 08041 Barcelona, Spain; 2 Grup de Neurofarmacologia Molecular, Institut de Neurociències, Universitat Autònoma de Barcelona, 08193 Barcelona, Spain

**Keywords:** anxiety, chemotherapy-induced peripheral neuropathy, depression, hydrogen rich water, inflammation, molecular hydrogen, oxidative stress, paclitaxel

## Abstract

Chemotherapy-provoked peripheral neuropathy and its linked comorbidities severely reduce the quality of a patient’s life. Its therapy is not completely resolved and has become an important clinical challenge. The protective actions of molecular hydrogen (H_2_) in many neurological disorders have been described, but its effects on memory and the emotional deficits accompanying neuropathic pain induced by chemotherapy remain unknown. In this study, using male mice injected with paclitaxel (PTX), we examined the effects of systemic treatment with hydrogen-rich water (HRW) in: (i) the mechanical and thermal allodynia provoked by PTX and the pathways involved; (ii) the memory deficits, anxiety- and depressive-like behaviors associated with PTX-induced peripheral neuropathy (PIPN); and (iii) the plasticity (p-extracellular signal-regulated protein kinase; p-ERK ½), nociceptive (p-protein kinase B, p-Akt), inflammatory (p-nuclear factor of kappa light polypeptide gene enhancer in B-cells inhibitor, alpha; p-IKBα), and oxidative (4-hydroxynonenal: 4-HNE) alterations provoked by PIPN in the prefrontal cortex (PFC). The results revealed: (1) the antiallodynic actions of HRW administered at one or two times per day during 7 and 3 consecutive days; (2) the participation of Kv7 potassium channels and the Nrf2-heme oxygenase 1-NAD(P)H: quinone oxidoreductase 1 pathway in the painkiller effects of HRW; (3) the inhibition of memory deficits and the anxiodepressive-like behaviors related with PIPN induced by HRW; and (4) the normalization of p-ERK ½, p-Akt and 4-HNE up-regulation and the activation of antioxidant enzymes produced by this treatment in PFC. This study proposes HRW as a possible effective and safe therapy for PIPN and its associated cognitive and emotional deficits.

## 1. Introduction

Chemotherapy-provoked neuropathic pain is one of the prevalent adverse effects in cancer patients and an important clinical challenge because it severely reduces the patient’s quality of life, and its therapy is not completely established [1,2]. Patients receiving chemotherapy also experience other symptoms, such as cognitive and affective disorders that can aggravate pain sensation and have a negative impact on the patient’s well-being [3,4].

Currently, new chemotherapeutic agents with various mechanisms of action are continually being developed. However, despite these advances, classical agents such as paclitaxel (PTX) are also currently used to treat different types of cancer (lung, breast, ovarian, pancreas, melanoma) [5,6]. However, despite its clear clinical benefits, PTX-induced peripheral neuropathy (PIPN) [7,8] that is accompanied by memory deficits, depression, and anxiety, which in some cases can induce reduced treatment and/or even its discontinuation [9,10]. These symptoms have also been demonstrated in preclinical pain models [11,12,13,14]. At present, therapies to reduce PIPN are limited, with modest efficacy and notable side effects [15]. Therefore, one objective of this study is to find a safe and effective treatment to alleviate PIPN and the associated cognitive and emotional disorders.

Molecular hydrogen (H_2_) is an emerging and promising therapy due to its antioxidant, anti-inflammatory, anti-apoptotic, and protective effects demonstrated in various cardiovascular, neurodegenerative, and diabetes disorders [16]. This gas is widely distributed throughout the central (CNS) and peripheral nervous system (PNS), easily crosses the blood–brain barrier and does not induce demonstrable side effects after its repetitive administration [17,18]. H_2_ also reversed the cognitive impairment observed in mouse models of Alzheimer’s [19] and in animals with acute ischemia-reperfusion injury [20]. The antidepressant and anxiolytic properties of this gas have also been described [21,22]. Moreover, several studies have demonstrated the anti-inflammatory properties of H_2_; in animals with osteoarthritis [23] and its antinociceptive effects in preclinical models of neuropathic pain caused by nerve injury [24,25,26]. A recent study further reveals that treatment with hydrogen-rich water (HRW) inhibits the affective disorders accompanying neuropathic pain caused by nerve damage [27]. Nevertheless, the possible modulatory action of HRW on the emotional disorders associated with PIPN has not been assessed. On the other hand, considering that memory deficits are difficult to restore once manifested and can persist for a long time, it is important to evaluate its possible inhibition with the administration of HRW to obtain a more-complete therapy against PIPN. Thus, in this research, the effects of treatment with HRW on the cognitive disorders linked with PIPN have been investigated.

It has been described that the voltage-gated Kv7 potassium channels play a crucial role in pain modulation [28,29] and that treatment with Kv7 channel activators have protective effects against chemotherapy-induced peripheral neuropathy [30]. Other works likewise proved the participation of these channels in the analgesic effects induced by some hydrogen sulfide (H_2_S) donors in animals with neuropathic pain occasioned by chemotherapy [31]. Otherwise, the protective role played by the nuclear factor erythroid 2-related factor 2 (Nrf2) pathway versus chemotherapy-induced oxidative stress has also been proved [32]. In this line, it is important to mention that most of the curative properties of several carbon monoxide (CO) or H_2_S donors during neuropathic pain provoked by nerve injury or chemotherapy do so by normalizing the down-regulation and/or increasing the expression of Nrf2 and/or several antioxidant enzymes [13,33]. In PTX-injected mice, we assessed the feasible participation of Kv7 potassium channels and the Nrf2-heme oxygenase 1 (HO-1)-NAD(P)H: quinone oxidoreductase 1 (NQO1) signaling pathway in the analgesic effects produced by HRW using specific blockers or inhibitors of these pathways.

The underlying mechanisms implicated in the progress and/or preservation of PIPN have not been completely elucidated. Some studies revealed that PTX provoked allodynia by inducing the phosphorylation of mitogen-activated protein kinase (MAPK) and of protein kinase B (Akt) [34]. Thereby, their inhibition prevented the development of PIPN [35,36]. In this research, we evaluated the effects of HRW on the p-Akt and p-ERK ½ levels in the prefrontal cortex (PFC) of animals injected with PTX. PFC is a brain-modulatory area of pain, cognitive and affective disorders [37,38].

PIPN is correlated with inflammatory processes [8]; thus, the increased expression of phosphorylated NF-kB was demonstrated in PTX-injected mice, and its inhibition protected against chemotherapy-induced neuropathy [39]. Other studies have also proved that the administration of PTX disrupts homeostasis, provoking apoptosis in the PNS and/or CNS [40]. In accordance with these findings, we examined the actions of HRW treatment in some proteins related to inflammatory (phosphorylated-NF-κB inhibitor alpha; p-IKBα) and apoptotic (BAX) processes in PFC of PTX-injected mice.

Other studies have also shown the up-regulation of different oxidative-stress-related proteins in the PFC of animals with PIPN and that its inhibition is involved in the palliate effects produced by H_2_S donors and HO-1 inducers [13]. In this research, we assessed the effects of HRW on the expression of the oxidative biomarker 4-hydroxynonenal (4-HNE) and the antioxidant enzymes HO-1, NQO1, glutathione S-transferase mu 1 (GSTM1) and superoxide dismutase 1 (SOD-1) in the PFC of mice with PIPN.

## 2. Materials and Methods

### 2.1. Animals

Male C57BL/6 mice (5–6-week-old and 21–25 g), acquired from Envigo Laboratories (Barcelona, Spain), were employed. The mice were maintained in regulated environmental conditions, 22 °C temperature, 66% humidity, and 12 h dark/light cycle, with food and water ad libitum. The experiments were started after 7 days to allow for the acclimatization of the housing conditions and performed between 9:00 a.m. and 5:00 p.m. The experiments were performed in agreement with the guidelines of the European Commission’s directive (2010/63/EC) and the Spanish Law (RD 53/2013) regulating animal research, and the procedure was approved by the local Committee of Animal Use and Care of the Autonomous University of Barcelona (ethical code 9863). Every effort was made to reduce the suffering and number of mice. 

### 2.2. PTX Treatment

Painful neuropathy was provoked with the intraperitoneal administration of 2 mg/kg of PTX every other day, for four successive days, in accordance with previous studies [11,13]. PTX acquired from Tocris Bioscience (Bristol, UK) was dissolved in a 1:1:18 mixture solution containing Cremophor EL (Sigma-Aldrich, St. Louis, MO, USA), ethanol and saline (SS, 0.9% NaCl). Control groups were injected with vehicle solution in a similar schedule.

### 2.3. Allodynia Tests

Mechanical allodynia was assessed by determining the hind paw withdrawal response following the stimulation using von Frey filaments of distinct bending forces (North Coast Medical, Inc., San Jose, CA, USA). Animals remained inside the methacrylate cylinders (20 cm high per 9 cm diameter) (Servei Estació, Barcelona, Spain), placed in a grid bottom through the filaments were applied in the hind paws in agreement with the up–down paradigm [41]. The test started with a filament of 0.4 g, and the stiffness of the following filament was chosen according to the response. An Excel program (Microsoft Iberia SRL, Barcelona, Spain), which included curve fitting of the data, was used to calculate the threshold of the response. Shaking, withdrawing, or licking the paw was recorded as a positive reaction.

To evaluate cold allodynia, a cold plate analgesiometer (Ugo Basile, Italy) at 4 ± 0.5 °C was used, and the number of paw elevations was recorded for 5 min.

In both tests, both hind paws were tested. 

### 2.4. Cognitive Behavior Test

The evaluation of the novel object recognition memory was performed in a gray box (44 × 44 cm) with four walls and a non-reflective base [42]. This test comprises four sessions of 10 min. On the first two days, the animals were habituated to the empty box. On day 3, the mice were put once again in the box and two equal objects were shown. At day 4, one of the familiar objects was changed to a new object. The time exploring the new and familiar objects was quantified. The discrimination index defined as ((time exploring the new object–time exploring the familiar object)/(time exploring the novel object + time exploring the familiar object) × 100) was utilized as a measure of cognitive behavior. A high discrimination index signifies good recognition memory.

### 2.5. Emotional Behavior Tests

The evaluation of depressive-like behaviors was performed by using the tail suspension test (TST) and the forced swimming test (FST). In both, the duration of immobility, as considered when the animals remained completely still, was quantified.

In the TST, the animals were suspended at 35 cm from the floor by using adhesive tape to the tip of the tail and fixing it to a surface. Their movements were filmed with a digital camera, and the immobility time was evaluated for 6 min [43].

In the FST, the mice were placed in methacrylate cylinders (25 cm high and 10 cm diameter) containing water, at 24 ± 2 °C, up to a 10 cm depth. Their activity was recorded with a digital camera for 6 min, and the time spent immobile during the last 4 min was registered [44].

The anxiety-like behaviors were assessed using the elevated plus maze (EPM) [45] and the open file (OF) tests [46]. 

The EPM apparatus used had 4 arms, two closed by 15 cm high walls and two open, elevated at 45 cm from the floor. Each of them was 5 cm wide and 35 cm long. The mice were always positioned in the center of the maze, facing the same open arm, and permitted to explore for 5 min. Their movements were filmed, and the number of entries into the closed and open arms, as well as the percentage of time passed in the open arms, were quantified. 

In the OF test, the mice were positioned in the center of a box of 44 cm × 44 cm, enclosed by gray walls (30 cm high), and allowed to explore it for 5 min. They were filmed by a digital camera, and the number of entries and the percentage of time spent in the central area, as well as the number of squares crossed, were evaluated.

In all tests, the animals were familiarized with the testing space for 1 h prior to starting the proof, and these experiments were performed by researchers blinded to the experimental conditions.

### 2.6. Western Blot

At 21 days after PTX or vehicle injection, the animals were euthanized with cervical dislocation. PFCs were removed, frozen in dry ice, and maintained at −80 °C. The sonication of tissues was performed in a cold lysis buffer RIPA Buffer (Sigma-Aldrich, MO, USA). After solubilization for 1 h at 4 °C, the crude homogenates were sonicated again for 10 s and centrifuged at 700 *g* for 20 min at 4 °C. The supernatant (60 μg of total protein) was mixed with 4 x Laemmli loading buffer and loaded onto 4% stacking/12% separating sodium dodecyl sulfate-polyacrylamide gels. Next, the proteins were transferred onto a polyvinylidene fluoride membrane by electrophoresis (120 min) and blocked with phosphate-buffered saline (PBS) + 5% nonfat dry milk, PBS with Tween 20 + 5% bovine serum albumin (BSA), or Tris-buffered saline plus Tween 20 + 5% of nonfat dry milk or BSA, for 75 min. Then, the membranes were incubated with specific rabbit primary antibodies anti-p-ERK ½ (1:250), ERK ½ (1:250), p-Akt (1:200), Akt (1:200), IKBα (1:200) and BAX (1:250) purchased at Cell Signaling Technology (Danvers, MA, United States); p-IKBα (1:200), 4-HNE (1:150) and HO-1 (1:250) acquired at Abcam (Cambridge, UK); GSTM1 (1:150) and SOD-1 (1:150) from Novus Biologic (Littleton, CO, USA); and NQO1 (1:250) and GAPDH (1:5000) from Merck (Billerica, MA, USA) overnight at 4 °C. After that, the blots were incubated with a horseradish peroxidase-conjugated anti-rabbit secondary antibody (GE Healthcare, Little Chalfont, UK), 1 h at room temperature, and the proteins were detected with chemiluminescence reagents (ECL kit; GE Healthcare, Little Chalfont, UK). Densitometric analysis was performed employing the Image-J program (National Institutes of Health, Bethesda, MD, USA).

### 2.7. Experimental Procedures

At first, in the PTX- and vehicle-injected mice, the mechanical and thermal antiallodynic effects induced by HRW or the vehicle administered at 1 time (1T) or 2 times (2T) per day during 7 consecutive days, from days 15 to 21 after PTX injection, were evaluated (*n* = 6 animals for each group). Considering the major analgesic effectiveness of HRW administered at 2T per day versus 1T per day, all of the following experiments were performed in animals treated with HRW at 2T per day.

To study the role played by Kv7 potassium channels and the Nrf2-HO-1-NQO1 pathways in the antiallodynic actions induced by HRW, both PTX- or vehicle-injected animals were treated with HRW or vehicle combined with the vehicle, XE-991 (a Kv7 potassium channels blocker), ML-385 (Nrf2 inhibitor) [47], tin protoporphyrin IX (SnPP; HO-1 inhibitor) [48] or dicoumarol (NQO1 inhibitor) [49] administered at 2T per day for 3 consecutive days (days 19 -21 post-PTX injection). Allodynia was assessed on each day of treatment (*n* = 6 animals for group).

The effects of HRW on memory deficits and depressive- and anxiety-like behaviors associated with PIPN were evaluated in different groups of vehicle- or PTX-injected mice treated with HRW, administered at 2T per day for 3 days (days 19 to 21 post-PTX injection). The behavioral tests were undertaken on the third day of HRW administration (21 days after PTX injection) (*n* = 8 animals for group).

Finally, the effects of HRW on the expression of p-ERK ½, p-Akt, p-IKBα, BAX, 4-HNE, HO-1, GSTM1, NQO1, and SOD-1 in the PFC were evaluated using Western blot (*n* = 3 samples for each group).

### 2.8. Drugs

A hydrogen water generator from Hydrogen (Osmo-star Soriano S.L., Alicante, Spain) was employed to prepare HRW, as described by [27]. The XE-991, acquired from Tocris Bioscience (Ellisville, MO, USA), SnPP from Frontier Scientific (Livchem GmbH & Co., Frankfurt, Germany), ML-385 and dicoumarol from Eurodiagnostico S.L. (Madrid, Spain) were dissolved in dimethyl sulfoxide (1% solution in SS). 

All of the drugs were intraperitoneally administered to a final volume of 10 ml/kg. HRW at 0.3 mM was administered 1 h before testing, whereas 12 µmols/kg of XE-991, 25 mg/kg of ML-385, or 10 mg/kg of SnPP or dicoumarol were administered 30 min before the tests, in conformity with another study [27]. All of the drugs were newly prepared before administration. For each group treated with a drug, the respective control group received the same volume of analogous vehicles.

### 2.9. Statistical Analyses

The results are expressed as the mean values ± standard error of the mean (SEM). The three-way repeated measures ANOVA with time, treatment, and injection as the variable factors, followed by the one-way ANOVA and Student–Newman–Keuls tests, were applied to evaluate the effects of HRW administered alone or combined with XE-991, ML-385, SnPP, or dicoumarol on PTX-induced allodynia. The actions of the HRW treatment on the cognitive and emotive behaviors, as well as in the expression of several proteins, were analyzed by using a one-way ANOVA followed by the Student–Newman–Keuls test. The statistical analysis was performed utilizing the SPSS (version 28, IBM, Madrid, Spain) and Prism 8.0 (Graphpad, La Jolla, CA, USA) programs. A value of *p* < 0.05 was considered statistically significant.

## 3. Results

### 3.1. The Mechanical and Cold Allodynia Provoked by PTX Were Inhibited by the Repetitive Administration of HRW

The three-way ANOVA repeated measures revealed significant effects of time, treatment, and injection and their interactions (*p* < 0.001) in both mechanical and thermal allodynia provoked by PTX in both paws. 

**Figure 1:**
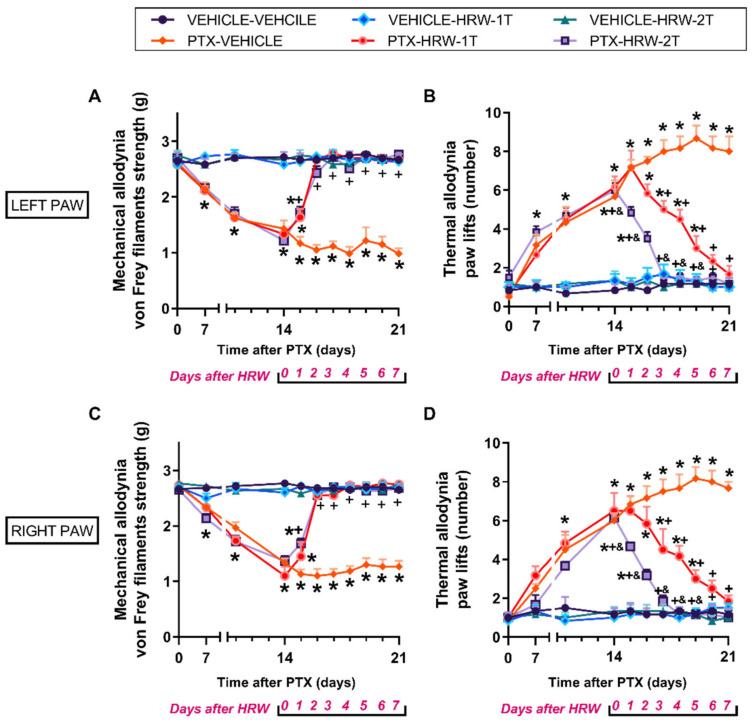
Effects of treatment with HRW on the mechanical and cold allodynia provoked by PTX. The mechanical (**A**,**C**) and thermal (**B**,**D**) antiallodynic effects induced by HRW, administered a 1T or 2T per day, during seven consecutive days from day 15 to 21 after vehicle or PTX administration, in the left (**A**,**B**) and right (**C**,**D**) hind paws are represented. In all graphs, for each day and treatment evaluated, * represents significant changes when compared to their respective vehicle-treated animals, + vs. PTX-vehicle-treated animals and & vs. PTX-HRW 1T per day treated animals (*p* < 0.05, one-way ANOVA followed by the Student–Newman–Keuls test). Results are shown as mean values ± SEM; *n* = 6 animals per experimental group.

In both hind paws, our results revealed that the mechanical allodynia provoked by PTX from day 14 to 21 after injection (*p* < 0.001; one-way ANOVA, against vehicle plus vehicle-treated animals; Figure 1A,C), was completely reversed by treatment with HRW administered at 1T and 2T per day during two consecutive days. The daily administration of HRW also progressively reduced the thermal allodynia induced by PTX injection (*p* < 0.001; one-way ANOVA, against vehicle plus vehicle-treated animals; Figure 1B,D), but five and three days of treatment with HRW, administered at 1T and 2T per day, were required for full inhibition.

In both tests and paws, treatment with HRW administered at 1T or 2T per day did not produce any effect in the vehicle-injected animals.

### 3.2. Reversion of the Antiallodynic Actions Induced by HRW Treatment with a Kv7 Potassium Channels Blocker and Specific Nrf2, HO-1 and NQO1 Inhibitors

To explore the role of Kv7 potassium channels and the antioxidant pathway triggered by the Nrf2 transcription factor in the antiallodynic effects of HRW, the reversion of its effects with specific blockers or inhibitors was evaluated.

**Figure 2:**
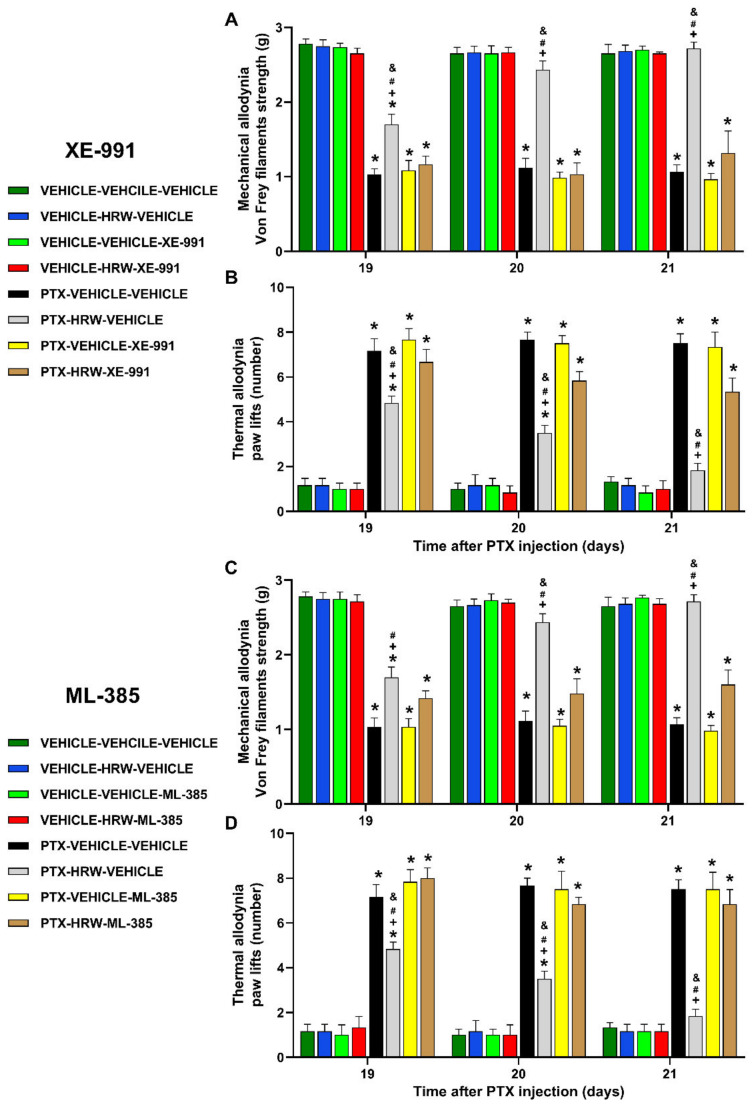
Reversion of the antiallodynic actions induced by HRW with the co-administration of XE-991 (a Kv7 potassium channels blocker) and ML-385 (a Nrf2 inhibitor) in the left hind paws of PTX-injected animals. The effects of the co-treatment of vehicle or HRW with XE-991 (**A**,**B**) or ML-385 (**C**,**D**), administered at 2T per day from days 19 to 21, on the mechanical (**A**,**C**) and thermal allodynia (**B**,**D**) provoked by PTX are represented. In all graphs, for each treatment and day analyzed, * reveals significant variations vs. their respective vehicle-injected animals, + vs. PTX plus vehicle-vehicle-treated animals, # vs. PTX plus vehicle-XE-991 or vehicle-ML-385 treated animals and & vs. PTX plus HRW-XE-991 or HRW-ML-385-treated mice (*p* < 0.05, one-way ANOVA and Student–Newman–Keuls test). Mean values ± SEM (*n* = 6 mice per group).

**Figure 3:**
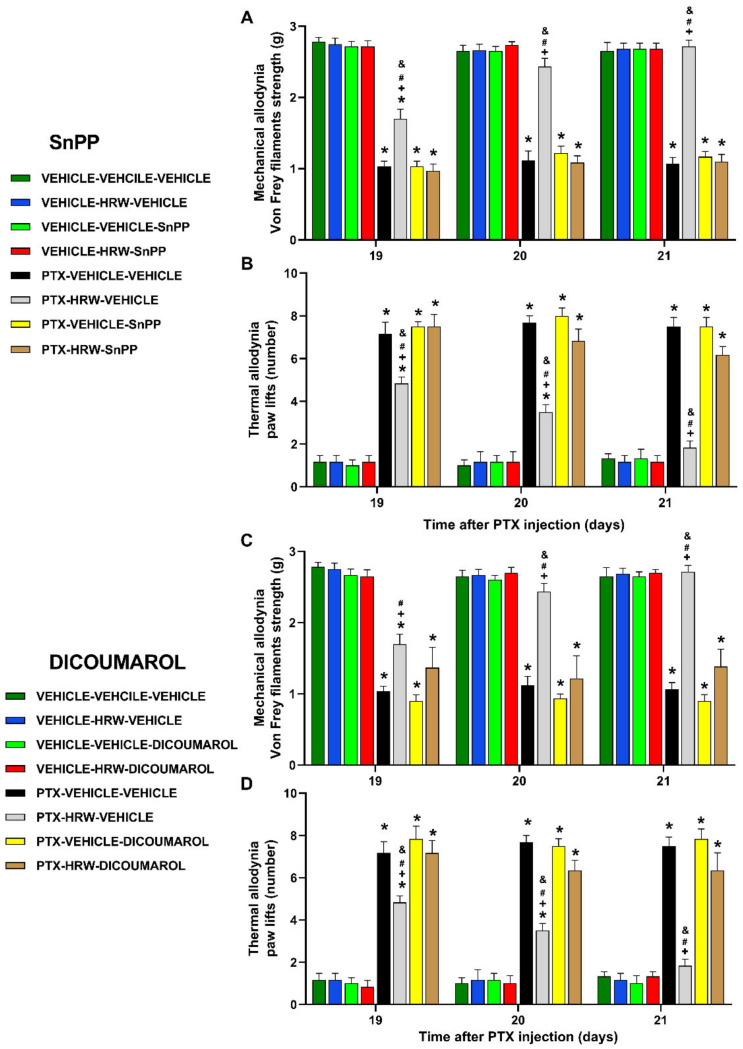
Reversal of the antiallodynic effects of HRW with the co-administration of SnPP (an HO-1 inhibitor) and dicoumarol (an NQO1 inhibitor) in the left hind paws of PTX-injected animals. The effects produced by the repetitive co-administration of vehicle or HRW with SnPP (**A**,**B**) or dicoumarol (**C**,**D**), administered at 2T per day from days 19 to 21, on the mechanical (**A**,**C**) and thermal allodynia (**B**,**D**) provoked by PTX are represented. In all pictures, for each treatment and day analyzed, * reveals significant differences vs. their respective vehicle-injected animals, + vs. PTX plus vehicle-vehicle-treated mice, # vs. PTX plus vehicle-SnPP or vehicle dicoumarol-treated mice and & vs. PTX plus HRW-SnPP or HRW-dicoumarol treated animals (*p* < 0.05, one-way ANOVA and Student–Newman–Keuls test). Mean values ± SEM (*n* = 6 animals per group).

In both hind paws, a three-way repeated measures ANOVA showed significant effects for the time, treatment, and injection (*p* < 0.001), as well as the interactions between them (*p* < 0.001), in the reversion of the mechanical and thermal antiallodynic effects of HRW induced by the administration of XE-991 (a Kv7 potassium channel blocker), ML-385, SnPP, and dicoumarol (specific Nrf2, HO-1, and NQO1 inhibitors). That is, our results demonstrated that the repetitive administration of XE-991 (Figure 2A,B), ML-385 (Figure 2C,D), SnPP (Figure 3A,B), or dicoumarol (Figure 3C,D) progressively reversed the mechanical and thermal antiallodynic effects of HRW. In all cases, while two days of treatment with the blocker or each inhibitor completely reversed the mechanical antiallodynic actions of HRW, the total reversion of the thermal antiallodynic effects of HRW required three days of treatment with XE-991, ML-385, SnPP, or dicoumarol (*p* < 0.001; one-way ANOVA, followed by the Student–Newman–Keuls test).

In all paradigms, treatment with XE-991, ML-385, SnPP, or dicumarol alone did not modify the mechanical and thermal allodynic responses provoked by PTX (Figure 2 and Figure 3). Similar results were observed in the right hind paws of mice injected with vehicle- or PTX (results not displayed).

### 3.3. HRW Treatment Inhibited the Cognitive and Emotional Disorders Associated with PIPN

**Figure 4:**
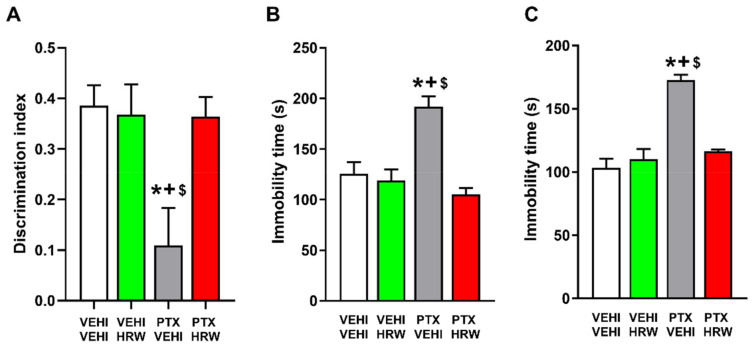
The effects of HRW on the memory deficits and depressive-like behaviors associated with PIPN. Effects of the repetitive administration of HRW or vehicle (VEHI) during 3 consecutive days on the memory deficits in the novel object recognition test (**A**), and depressive-like behaviors in the TST (**B**) and FST (**C**) accompanying PIPN. The discrimination index obtained in the novel object recognition test (**A**) and the immobility time(s) in the TST (**B**) and FST (**C**) are represented. In all panels, * indicates significant differences vs. VEHI plus VEHI treated animals; + vs. VEHI plus HRW treated animals and $ vs. PTX plus HRW-treated animals (*p* < 0.05, one-way ANOVA followed by the Student–Newman Keuls test). Data are expressed as mean values ± SEM; *n* = 8 animals per group.

Our data confirmed the cognitive deficits (Figure 4A) and depressive-like behaviors (Figure 4 B,C) associated with PIPN. Our results further revealed that the double administration of HRW per day, during 3 consecutive days, normalized the decreased discrimination index observed in PTX-injected animals in the novel object recognition test (*p* < 0.001; one-way ANOVA and Student–Newman–Keuls test; Figure 4A) and the augmented immobility time detected in PTX–injected mice in the TST (*p* < 0.001, one-way ANOVA and Student–Newman–Keuls test; Figure 4B) and FST (*p* < 0.001; one-way ANOVA and Student–Newman–Keuls test; Figure 4C). Thus, showing the recovery of memory deficits and the antidepressant effects produced by HRW treatment in animals with PIPN.

**Figure 5:**
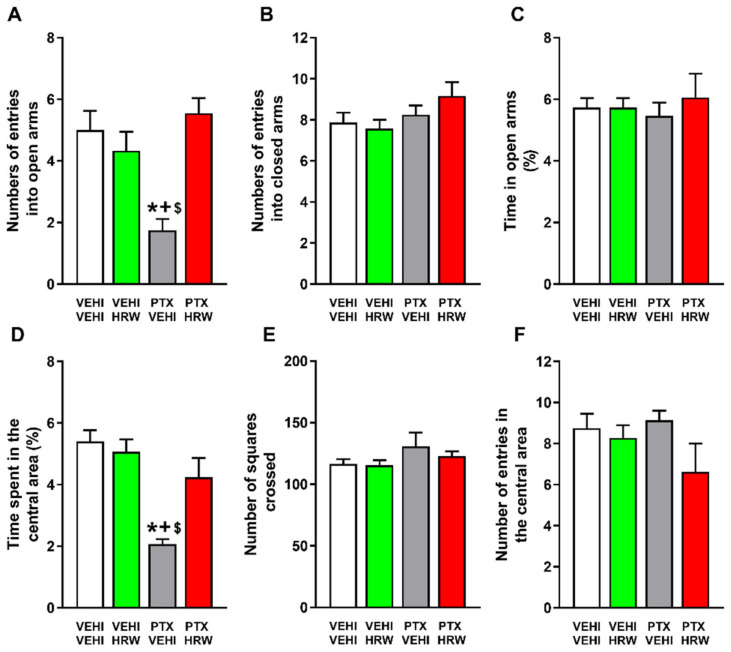
The effects of HRW on the anxiety-like behaviors linked with PIPN. Effects of the repetitive administration of HRW or vehicle (VEHI), during three consecutive days, on the anxiety like-behaviors accompanying PIPN. In the EPM test, the number of times that mice entered to the open arms (**A**), the quantity of entrances into the closed arms (**B**) and the the percentage of time spent in the open arms (**C**), are represented. In the OF test, the percentage of time spent within the central area (**D**), the total number of squares crossed (**E**) and the number of entries into the central area (**F**) are shown. In all panels, * denotes significant changes vs. VEHI plus VEHI treated animals; + respect to VEHI plus HRW treated animals and $ respect to PTX plus HRW-treated mice (*p* < 0.05, one-way ANOVA and Student–Newman–Keuls test). Results are represented as mean values ± SEM; *n* = 8 animals per group.

The anxiety-like behaviors previously observed in PTX-injected mice were supported by the diminished number of entrances into the open arms (*p* < 0.001, one-way ANOVA test with respect to the vehicle plus vehicle-treated mice; Figure 5A) and the reduced time spent in the central area (*p* < 0.001, one-way ANOVA test with respect to the vehicle plus vehicle-treated mice; Figure 5D) of the EPM and OF tests. Our results further showed that the double daily administration of HRW normalized both diminished responses, thus revealing the anxiolytic effects of this treatment. The non-changes in the number of entries into the closed arms of the EPM (Figure 5B), nor in the number of squares crossed in the OF test (Figure 5E) observed in the HRW-treated animals substantiated the absence of changes in the locomotor activity of these animals. Non-alterations in the percentage of time spent in the open arms of the EPM (Figure 5C) nor in the number of entries in the central area of the OF (Figure 5F) were detected.

### 3.4. Effects of Treatment with HRW on the Expression of p-ERK ½, p-Akt, p-IKBα and BAX in the PFC of PTX-Injected Mice

**Figure 6:**
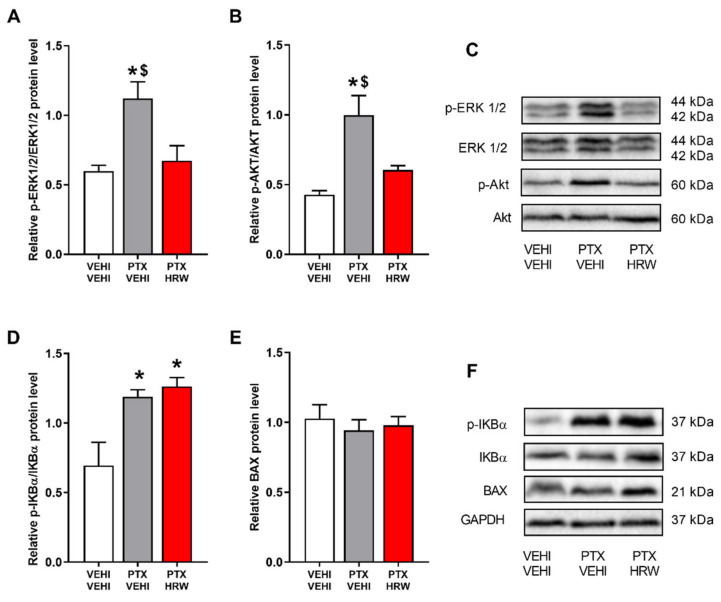
The effects of HRW on the p-ERK ½, p-Akt, p-IKBα, and BAX expression in the PFC of PTX-injected animals. The protein levels of p-ERK ½/ERK ½ (**A**), p-Akt/Akt (**B**), p-IKBα/IKBα (**D**) and BAX (**E**) in the PFC of PTX-injected mice treated with HRW are shown. Control animals treated with vehicle (VEHI) plus VEHI are also displayed. Representative blots for p-ERK ½, ERK ½, p-Akt, and Akt (**C**), p-IKBα, IKBα, BAX and GAPDH (**F**) are presented. In all graphs, * denotes significant differences with respect to VEHI plus VEHI-treated mice and $ with respect to PTX plus HRW-treated mice (*p* < 0.05, one-way ANOVA and Student–Newman–Keuls test). Results are represented as mean values ± SEM (*n* = 3 samples per group).

Our findings demonstrated that PTX stimulated the phosphorylation of ERK ½ (*p* < 0.017, one-way ANOVA and Student–Newman–Keuls test versus vehicle plus vehicle-treated animals; Figure 6A), Akt (*p* < 0.008, one-way ANOVA and Student–Newman–Keuls vs. vehicle plus the vehicle-treated animals; Figure 6B), and IKBα (*p* < 0.010, one-way ANOVA and Student–Newman–Keuls vs. vehicle plus vehicle-treated animals; Figure 6D). The results further demonstrated that the HRW treatment normalized p-ERK ½ and p-AKT overexpression but did not modify the PTX-induced p-IKBα upregulation. Thus, showing the inhibition of the plasticity and nociceptive changes induced by HRW treatment in the PFC of PTX-injected mice. Non-changes in the protein levels of BAX were observed (Figure 6E). 

### 3.5. Effects of Treatment with HRW on the Expression of 4-HNE, HO-1, GSTM1, NQO1 and SOD-1 in the PFC of PTX-Injected Mice

Considering the relevant participation of oxidative stress in the development of PIPN, the effects of treatment with HRW in the expression of 4-HNE and the antioxidant proteins HO-1, GSTM1, NQO1, and SOD-1 in the PFC of PTX-injected mice were evaluated.

**Figure 7:**
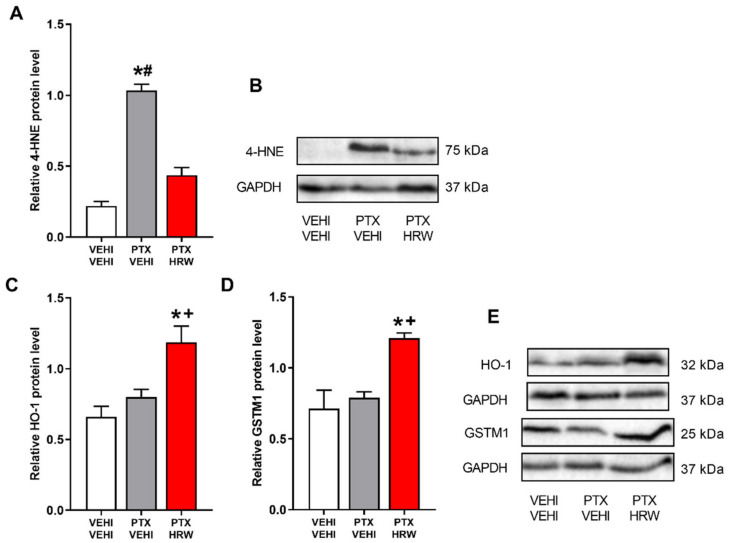

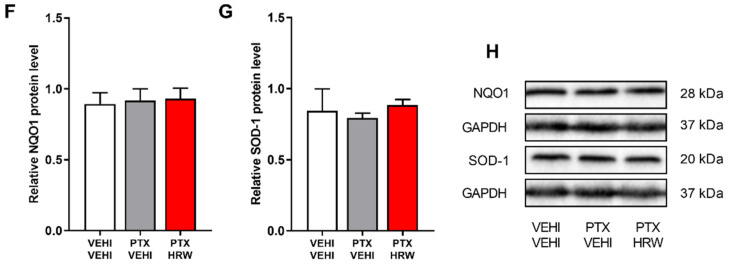
Effects of treatment with HRW on the expression of 4-HNE, HO-1, GSTM1, NQO1 and SOD-1 in the PFC of animals injected with PTX. The protein levels of 4-HNE (**A**), HO-1 (**C**), GSTM1 (**D**), NQO1 (**F**), and SOD-1 (**G**) in the PFC of PTX-injected mice treated with HRW are shown. Control animals treated with vehicle (VEHI) plus VEHI are also displayed. Representative blots of 4-HNE and GAPDH (**B**), HO-1, GSTM1 and GAPDH (**E**) and NQO1, SOD-1 and GAPDH (**H**) are presented. In all panels, * indicates significant differences vs. the VEHI plus VEHI-treated mice, + indicates significant differences vs. the PTX plus VEHI-treated mice, and #, vs. PTX plus HRW-treated mice (*p* < 0.05, one-way ANOVA followed by the Student–Newman–Keuls test). Data are expressed as mean values ± SEM; *n* = 3 samples per group.

Oxidative stress was confirmed by the increased expression of 4-HNE as compared with vehicle plus vehicle-treated mice (*p* < 0.001, one-way ANOVA and Student–Newman–Keuls; Figure 7A). Interestingly, treatment with HRW, in addition to normalizing 4-HNE overexpression, also augmented the levels of the antioxidant enzymes HO-1 (*p* < 0.011; ANOVA and Student–Newman–Keuls vs. vehicle plus vehicle and PTX plus vehicle-treated animals; Figure 7C) and GSTM1 (*p* < 0.010; ANOVA followed by Student–Newman–Keuls vs. vehicle plus vehicle and PTX plus vehicle-treated animals; Figure 7D). Thus, indicating the antioxidant properties of HRW in the PFC of PTX-injected animals. 

Regarding the protein levels of NQO1 (Figure 7F) and SOD-1 (Figure 7G), no significant changes were observed in PTX-injected mice treated with vehicle or HRW.

## 4. Discussion

This study demonstrated the inhibition of the allodynia, cognitive impairment and anxious–depressive-like behaviors induced by the repetitive treatment with HRW in mice with PIPN and revealed the likely pathways involved in these actions using pharmacological and molecular approaches.

Our results showed that the repetitive treatment with HRW, administered at 1T or 2T per day, reduced the mechanical and thermal allodynia provoked by PTX in both hind paws. These data evidenced, for the first time, the antiallodynic actions of HRW treatment in mice with PIPN. Our findings further showed that the double administration of HRW per day was more effective than the simple. Indeed, the PTX-induced allodynia was completely inhibited after three and seven days of treatment with HRW administered at 2T or 1T per day. These findings agree with the inhibitory actions produced by HRW or hydrogen-rich saline in mice with nerve injury-induced neuropathic pain [24,25,26,27], with the prevention of remifentanil-provoked post-surgical hyperalgesia [50] and with the improved hyperalgesia induced by oxaliplatin [51]. Our results also supported the major effectiveness produced by the double daily administration of HRW versus the simple as recently demonstrated in animals with neuropathic pain provoked by nerve injury [27].

Regarding the main pathways involved in the analgesic actions of HRW in animals with PIPN, we evaluated the possible participation of Kv7 potassium channels by assessing the reversion of HRW effects with the co-treatment with the selective Kv7 potassium channels blocker, XE-991. Our results showed that the inhibition of the mechanical and thermal allodynia produced by HRW was reversed with the administration of XE-991. These data agreed with the reversion of the antinociceptive effects produced by several H_2_S donors with the administration of Kv7 potassium channels blockers during chronic pain [31] and further revealed the participation of these channels in the pain-relieving actions of HRW in mice with PIPN.

Oher works showed the crucial role played by the Nrf2 transcription factor in the effects produced by H_2_ in different experimental conditions [18,52]. In this study, we assessed the importance of this pathway in the painkilling actions of HRW in mice with PIPN. The reversal of the analgesic effects of HRW with the co-administration with ML-385, SnPP, or dicoumarol revealed the involvement of the antioxidant system in the inhibition of the mechanical and thermal allodynia produced by HRW in animals with PIPN. These data supported other studies made with H_2_ [27] or H_2_S donors, which also activated the Nrf2/HO-1 and/or NQO1 path for inhibiting nerve injury-induced neuropathic pain in rodents [33].

This work confirmed the memory deficits caused by PTX, as previously demonstrated by the reduced discrimination index in the novel object recognition test [14], and further proved that the administration of HRW normalized this cognitive impairment. These results are in accordance with the prevention of stress-induced decline of memory produced by the continuous consumption of HRW in mice [53], with the efficacy of other gases in inhibiting the memory deficits associated with chronic osteoarthritis pain [54] as well as with those produced by 7-chloro-4-(phenylselanyl) quinoline in animals with PIPN [55]. Several works indicated the contribution of the plasticity changes in memory impairments and that several drugs can improve these deficits by restoring the altered expression of MAPK activated by chemotherapy in the CNS [14]. The reversion of the increased expression of p-ERK ½ caused by PTX with the HRW treatment agreed with the effects produced by this treatment in the PFC of sciatic nerve-injured mice [27] and further suggested that the normalization of the plasticity changes produced by HRW might be involved in the recovery of memory deficits produced by this treatment in PTX-injected mice.

In accordance with a recent publication [27], our data revealed the antidepressant effects of HRW in animals with PIPN. In compliance, another work showed that HRW could prevent chronic stress-induced depressive-like behaviors in mice [21]. Even so, our results showed, for the first time, the therapeutic potential of HRW against the depressive-like behaviors associated with PIPN. Moreover, considering that classic antidepressants produce a wide range of side effects and that treatment with HRW is considered a safe treatment with non-demonstrable relevant secondary effects [18], the therapeutic administration of HRW might be considered an effective alternative for treating the depressive-like behaviors linked with PIPN.

Our data further confirmed the anxiolytic-like behaviors related to PIPN [11,13] and revealed the anxiolytic properties of treatment with HRW in this pain model. Indeed, HRW reversed the low number of entries into the open arms and the low time passed in the central area of the EPM and OF tests, as displayed by PTX-injected mice treated with vehicles. The non-alterations in the locomotor activity induced by HRW were demonstrated by the absence of changes in the number of entries into the closed arms and in the number of squares traversed in the EPM and OF tests, respectively. Thus, indicating that the anxiolytic actions of HRW were not affected by possible locomotory alterations. These data concur with the anxiolytic properties of HRW in animals with neuropathic pain generated by nerve injury [27]. 

One of the multiple mechanisms underlying the affective disorders associated with PIPN is oxidative stress [13,56]. Moreover, the antidepressant and anxiolytic actions induced by several compounds are mainly produced by inhibiting oxidative stress [13,27,33]. In accordance, the oxidative-stress responses induced by PTX demonstrated by the increased expression of 4-HNE in PFC [13] and its normalization with HRW treatment revealed the antioxidative effects of H_2_ in the CNS of mice with PIPN. Accordingly, HRW also reversed the oxidative stress caused by oxaliplatin [51]. Our data also revealed an improvement in the HO-1 and GSTM1 expression in the PFC of HRW-treated animals as occurs with CoPP treatment [13]. Moreover, considering the antidepressant and anxiolytic properties of several antioxidant compounds, our results suggested that the antioxidant actions of HRW might be involved in the inhibition of the emotional disorders linked with PIPN. Nevertheless, because p-ERK ½ also takes part in the manifestation of the anxiodepressive-like behaviors by triggering inflammatory and oxidative responses in the cortex, and its inhibition avoided the affective alterations accompanying chronic pain [57], the stabilization of p-ERK ½ upregulation produced by HRW in PFC might also be engaged in the inhibition of the anxious-depressive-like behaviors linked with PIPN. Lastly, the non-changes in BAX expression observed in the PFC of animals injected with PTX alone or combined with HRW revealed that the apoptotic responses provoked by this chemotherapeutic agent in other brain areas, such as the hippocampus [58], are not observed in PFC [13].

In recent years, several works have investigated different treatments for preventing or treating chemotherapy-induced neuropathic pain, such as the administration of channel and neurotransmitter modulators, neuroprotectors, antidepressants, anticonvulsants, or opioids [59]. However, most of these therapies exhibited moderate efficacy and significant side effects such as somnolence, constipation, tolerance, and dizziness [60]. In addition, some treatments only improved neuropathy or one of the emotional disorders associated with chemotherapy-induced neuropathic pain [61,62,63], but only a few studies evaluated the possible effects of different treatments in the management of PIPN-associated mood disorders [13,64,65]. In this line, this study demonstrated that treatment with HRW inhibited neuropathy and avoided the cognitive and emotional deficits associated. Moreover, because the main nociceptive, cognitive, and emotional symptoms observed in our animal model are very similar to those observed in patients receiving chemotherapy [9], considering the few side effects induced by HRW, this treatment might be proposed as a safe and effective therapy against PIPN and the comorbidities associated.

## 5. Conclusions

In summary, this research demonstrates that the repetitive administration of HRW inhibited allodynia, memory deficits, and anxious-depressive-like behaviors provoked by PTX. This treatment normalized the plasticity changes, nociceptive responses and oxidative stress provoked by PTX and improved the expression of antioxidant enzymes, HO-1 and GSTM1, in the PFC of mice with PIPN. In summary, this work suggests that HRW treatment might be a good approach for the management of PIPN and its accompanying cognitive and emotive disorders.

## Data Availability

Data is contained within the article.
